# Treatment-Free Survival: A Novel Outcome Measure of the Effects of Immune Checkpoint Inhibition—A Pooled Analysis of Patients With Advanced Melanoma

**DOI:** 10.1200/JCO.19.00345

**Published:** 2019-09-09

**Authors:** Meredith M. Regan, Lillian Werner, Sumati Rao, Komal Gupte-Singh, F. Stephen Hodi, John M. Kirkwood, Harriet M. Kluger, James Larkin, Michael A. Postow, Corey Ritchings, Mario Sznol, Ahmad A. Tarhini, Jedd D. Wolchok, Michael B. Atkins, David F. McDermott

**Affiliations:** ^1^ Dana-Farber Cancer Institute, Boston, MA; ^2^ Harvard Medical School, Boston, MA; ^3^ Bristol-Myers Squibb, Princeton, NJ; ^4^ University of Pittsburgh Cancer Institute, Pittsburgh, PA; ^5^ Yale Cancer Center, New Haven, CT; ^6^ The Royal Marsden NHS Foundation Trust, London, United Kingdom; ^7^ Memorial Sloan Kettering Cancer Center, New York, NY; ^8^ Weill Cornell Medical College, New York, NY; ^9^ Yale University School of Medicine, New Haven, CT; ^10^ Emory University and Winship Comprehensive Cancer Center, Atlanta, GA; ^11^ Georgetown Lombardi Comprehensive Cancer Center, Washington, DC; ^12^ Beth Israel Deaconess Medical Center, Boston, MA

## Abstract

**PURPOSE:**

Outcome measures that comprehensively capture attributes of immuno-oncology agents, including prolonged treatment-free time and persistent treatment-related adverse events (TRAEs), are needed to complement conventional survival end points.

**METHODS:**

We pooled data from the CheckMate 067 and 069 clinical trials of nivolumab and ipilimumab, as monotherapies or in combination, for patients with advanced melanoma. Treatment-free survival (TFS) was defined as the area between Kaplan-Meier curves for two conventional time-to-event end points, each defined from random assignment: time to immune checkpoint inhibitor (ICI) protocol therapy cessation and time to subsequent systemic therapy initiation or death. TFS was partitioned as time with and without toxicity by a third end point, time to cessation of both ICI therapy and toxicity. Toxicity included persistent and late-onset grade 3 or higher TRAEs. The area under each Kaplan-Meier curve was estimated by the 36-month restricted mean time.

**RESULTS:**

At 36 months, many of the 1,077 patients who initiated ICI therapy were surviving free of subsequent therapy initiation (47% nivolumab plus ipilimumab, 37% nivolumab, 15% ipilimumab). The restricted mean TFS was longer for nivolumab plus ipilimumab (11.1 months) compared with nivolumab (4.6 months; difference, 6.5 months; 95% CI, 5.0 to 8.0 months) or ipilimumab (8.7 months; difference, 2.4 months; 95% CI, 0.8 to 4.1 months); restricted mean TFS represented 31% (3% with and 28% without toxicity), 13% (1% and 11%), and 24% (less than 1% and 23%) of the 36-month period, respectively, in the three treatment groups. TFS without toxicity was longer for nivolumab plus ipilimumab than nivolumab (difference, 6.0 months) or ipilimumab (difference, 1.7 months).

**CONCLUSION:**

The analysis of TFS between ICI cessation and subsequent therapy initiation revealed longer TFS without toxicity for patients with advanced melanoma who received nivolumab plus ipilimumab compared with nivolumab or ipilimumab. Regardless of treatment, a small proportion of the TFS involved grade 3 or higher TRAEs.

## INTRODUCTION

Immune checkpoint inhibitors (ICIs) produce unique patterns of antitumor response^1^ and toxicity.^2^ Although assessments of progression-free survival (PFS) and overall survival (OS) have guided regulatory approvals and clinical decisions, these conventional end points may not comprehensively assess outcomes with ICIs. Patients who discontinue ICIs may experience periods of remission or durable disease control without the need for subsequent systemic therapy.^3,4^ In patients with advanced melanoma treated with combination nivolumab plus ipilimumab in the randomized, double-blind, phase III CheckMate 067^5,6^ (ClinicalTrials.gov identifier: NCT01844505) and phase II CheckMate 069^7,8^ (ClinicalTrials.gov identifier: NCT01927419) trials, a subset discontinued nivolumab plus ipilimumab early because of treatment-related adverse events (TRAEs) and experienced durable responses, with a median time to subsequent systemic therapy of more than 2 years.^9^ These adverse events (AEs) also may persist or appear after ICI discontinuation.^2^ Because combination immunotherapies increasingly are used to enhance efficacy, the development of outcome measures that provide a full assessment of their benefits and consequences should be a priority.

Investigators traditionally have reported complementary analyses of the duration of response and treatment-free intervals, which are defined by the time from end of therapy until the need for next-line therapy, using classic time-to-event end point analyses (eg, Kaplan-Meier curves) and graphical patient histories (ie, swimmer plots).^3,8-10^ These analyses and plots typically report selected subsets of patients (eg, only those having response to therapy or discontinuing early because of TRAEs) and therefore represent a partial representation of the study population. Furthermore, the selected patients’ ongoing and/or delayed toxicity experience is not routinely incorporated.

We aimed to develop an outcome measure to characterize the time free of systemic anticancer therapy that may be achieved with ICIs. We included all patients who initiate therapy, rather than a selected subset of patients, and incorporated the possibility of persistent and/or late adverse effects of initial therapy to describe more completely the experience of every patient. An analysis of patients enrolled in CheckMate 067 and 069 allowed us to propose a novel outcome, treatment-free survival (TFS), that can characterize antitumor activity and be partitioned to include toxicity experienced during the period after cessation of ICI protocol therapy until initiation of subsequent systemic therapy or death.

## METHODS

### Study Design and Patients

We pooled data from 1,087 patients enrolled in two randomized, double-blind trials of nivolumab and ipilimumab (CheckMate 067 and 069), used in combination and as monotherapy, for previously untreated advanced melanoma.^6,8^ Study protocols were approved by the institutional review board at each participating study site, and all patients provided written informed consent before enrollment. The CheckMate 067 and 069 trials were sponsored by Bristol-Myers Squibb and were conducted in accordance with the Declaration of Helsinki and with Good Clinical Practice guidelines as defined by the International Conference on Harmonisation.

CheckMate 067 was a phase III study of 945 treatment-naïve patients with unresectable stage III or IV melanoma. Patients were randomly assigned in a 1:1:1 ratio to receive nivolumab 1 mg/kg plus ipilimumab 3 mg/kg every 3 weeks for four doses, followed by nivolumab 3 mg/kg every 2 weeks thereafter; nivolumab 3 mg/kg every 2 weeks (plus ipilimumab-matched placebo); or ipilimumab 3 mg/kg every 3 weeks for four doses (plus nivolumab-matched placebo) followed by placebo infusion every 2 weeks thereafter.

CheckMate 069 was a phase II study of 142 treatment-naïve patients with unresectable stage III or IV melanoma. Patients were randomly assigned in a 2:1 ratio to receive nivolumab 1 mg/kg plus ipilimumab 3 mg/kg by intravenous infusion every 3 weeks for four doses, followed by nivolumab 3 mg/kg every 2 weeks, or to receive ipilimumab 3 mg/kg with placebo every 3 weeks for four doses, followed by placebo infusion every 2 weeks.

In each trial, assigned ICI protocol treatment was continued until occurrence of progressive disease, unacceptable toxicity, or patient decision. Subsequent therapies were collected after protocol therapy discontinuation, except in the case of withdrawn consent. The minimum follow-up for this analysis was 3 years in both trials,^6,8^ despite the recently updated follow-up of CheckMate 067,^10^ to maintain consistent follow-up.

### End Points and Statistical Considerations

The analysis population included the 1,077 of 1,087 patients who initiated protocol therapy. The Kaplan-Meier method was used to estimate time-to-event end point distributions and the 36-month time to event.

**FIG 1.**
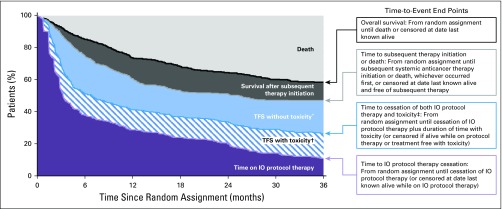
Illustration of the end points that partition the area under the overall survival curve into treatment-free survival (TFS) and other resulting health states. (*) Time after cessation of immuno-oncology (IO) protocol therapy without toxicity before initiation of subsequent systemic anticancer therapy or death. (†) Time after cessation of IO protocol therapy with toxicity while treatment free. (‡) Includes toxicity that persisted since protocol therapy and toxicity that newly presented after protocol therapy cessation.

TFS was based on the entire population and defined as the area between Kaplan-Meier curves for two fundamental time-to-event end points (Fig 1): time to ICI protocol therapy cessation, defined from random assignment until cessation of ICI protocol therapy or censored at date last known alive on protocol therapy, and time to subsequent therapy initiation or death, defined from random assignment until initiation of subsequent systemic anticancer therapy or death, whichever occurred first, or censored at date last known alive and free of subsequent therapy. TFS was estimated as the difference between the restricted mean event times^11^ of the two end points (ie, restricted mean time to subsequent therapy initiation or death minus restricted mean time to ICI protocol therapy cessation).

More comprehensively, we used the two end points to partition the area under the OS curve into three health states (Fig 1): time on ICI protocol therapy, TFS, and survival after subsequent therapy initiation. Each health state was characterized as an area between Kaplan-Meier curves and estimated as differences between restricted mean event times.^12,13^ OS was defined from random assignment until death as a result of any cause or was censored on the date last known alive.

TFS was partitioned further into states with and without toxicity by introducing another end point: time to cessation of both ICI protocol therapy and toxicity. The end point was calculated as the sum of the time to ICI protocol therapy cessation plus the number of days after cessation with an event-defining toxicity. We used three alternative event definitions for toxicity: days with a grade 3 or higher TRAE that either was persisting from the ICI protocol therapy or was newly reported after ICI discontinuation but before subsequent therapy initiation; days with a grade 2 or higher TRAE; and days when systemic or topical immunomodulatory medication was used for a TRAE of any grade after the ICI protocol therapy had been discontinued, with the exclusion of immunomodulatory or thyroxine-like medication use as hormone replacement for pituitary and adrenal insufficiency.

For the TFS analysis, time was restricted at 36 months since random assignment which was selected on the basis of the minimum available follow-up duration of the trials. The restricted mean time of each health state also was quantified as a percentage of the 36-month period. Between-group comparisons were based on the estimated between-group differences in restricted mean survival times, with bootstrapped 95% CIs.^12^

In enhanced swimmer plots that complemented the TFS analysis, treatment-free interval was defined for each individual patient who ceased ICI protocol therapy as the difference between the two end point values (ie, time to subsequent therapy initiation or death minus time to ICI protocol therapy cessation). Treatment-free interval was censored at 36 months since random assignment in patients who were alive and free of subsequent therapy after cessation of ICI protocol therapy.

## RESULTS

In this analysis, 999 of 1,077 patients who initiated ICI protocol therapy had ceased therapy, 499 of 1,077 patients had initiated subsequent systemic anticancer therapy, and 563 of 1,077 patients died (Data Supplement). The Kaplan-Meier estimates of OS at 36 months since random assignment in the pooled nivolumab plus ipilimumab, nivolumab, and ipilimumab groups were 58%, 52%, and 36% of patients, respectively (Data Supplement). The estimates of survival free of subsequent systemic anticancer therapy at 36 months were 47%, 37%, and 15% of patients, and 11%, 17%, and 0% of patients remained on ICI protocol therapy, respectively (Data Supplement).

**FIG 2.**
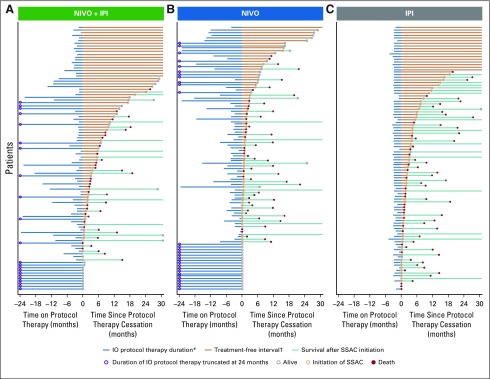
Swimmer plots of treatment-free interval between immuno-oncology (IO) protocol therapy cessation and subsequent systemic anticancer (SSAC) therapy initiation for individual patients with advanced melanoma in the CheckMate 067 and 069 trials. Plotted is a random sample of 100 patients per treatment group for (A) nivolumab (NIVO) plus ipilimumab (IPI), (B) NIVO, and (C) IPI. The *x*-axis is oriented relative to cessation of IO protocol therapy and is truncated at 24 months before and 30 months after cessation. (*) Patients who remained on IO protocol therapy had therapy duration censored at the origin. (†) Treatment-free intervals were highlighted by setting the origin of the *x*-axis as the point of IO protocol therapy cessation and sorting the individual patients by duration of the treatment-free interval.

As a preliminary descriptive summary of the observed treatment-free intervals, enhanced swimmer plots illustrated individual patients’ patterns of ICI protocol therapy duration, treatment-free interval, initiation of subsequent systemic anticancer therapy, and death (Fig 2). To facilitate visualization of details, representative, randomly selected subsets of 100 individuals were plotted per treatment group. Treatment-free intervals were highlighted by setting the *x*-axis origin as the point of ICI protocol therapy cessation and sorting individuals by duration of the treatment-free interval. Those patients who remained on ICI protocol therapy are shown by therapy duration censored at the origin.

**FIG 3.**
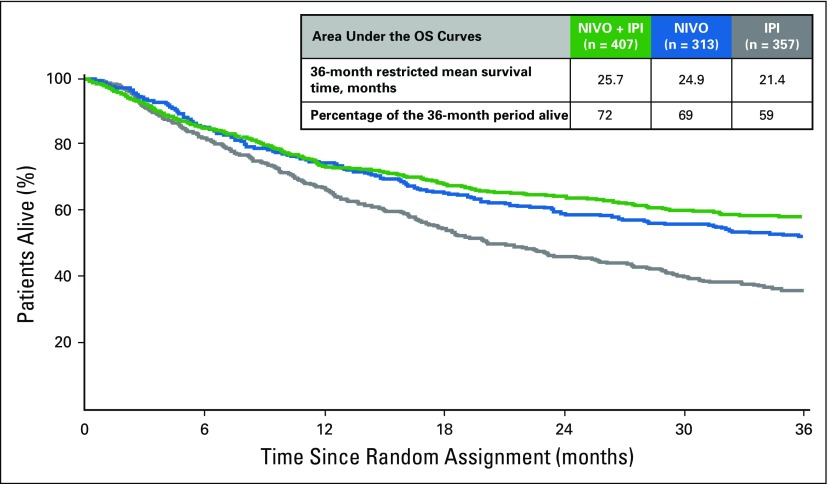
Kaplan-Meier estimates of overall survival (OS) over the 36-month period since random assignment and restricted mean OS according to treatment group. IPI, ipilimumab; NIVO, nivolumab.

**FIG 4.**
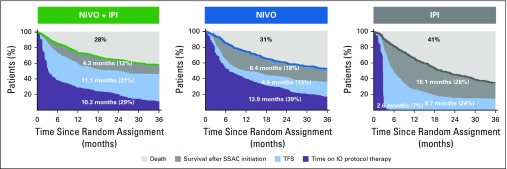
Estimates of treatment-free survival (TFS) and other health states over the 36-month period since random assignment according to treatment group. Data labels represent the mean number of months in any health state and the percentage of time in the 36-month period. IO, immuno-oncology; IPI, ipilimumab; NIVO, nivolumab; SSAC, subsequent systemic anticancer therapy.

The summary and comparison of these patterns in the population derive from partitioning the area under the OS curves for each treatment group. The restricted mean OS times were longer for the nivolumab plus ipilimumab and nivolumab groups than for the ipilimumab group; on average, patients survived 25.7, 24.9, and 21.4 months, respectively, of the 36-month period (Fig 3), which represented 72%, 69%, and 59% of the 36-month period alive. On average, the nivolumab plus ipilimumab, nivolumab, and ipilimumab groups spent 31%, 13%, and 24%, respectively, of the 36-month period alive and treatment free (Fig 4). Restricted mean TFS was 11.1 months with nivolumab plus ipilimumab *v* 4.6 months with nivolumab (difference, 6.5 months; 95% CI, 5.0 to 8.0 months) and 8.7 months with ipilimumab (difference, 2.4 months; 95% CI, 0.8 to 4.1 months; Data Supplement). The estimated TFS reflected that the nivolumab group had the longest ICI protocol therapy duration (restricted mean, 13.9 months; 39% of the 36-month period), and the ipilimumab group had the shortest protocol therapy duration by design (restricted mean, 2.6 months; 7% of the 36-month period) and longest survival after subsequent therapy initiation (restricted mean, 10.1 months; 28% of the 36-month period). The results were consistent between trials (Data Supplement).

**FIG 5.**
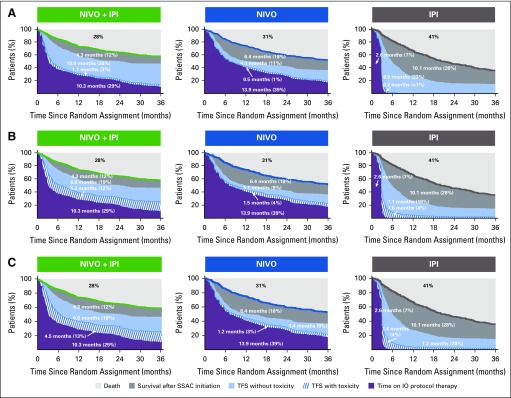
Estimates of treatment-free survival (TFS) with and without toxicity and other health states over the 36-month period since random assignment, according to treatment group. Toxicity is defined alternatively by (A) grade 3 or higher treatment-related adverse events (TRAEs), (B) grade 2 or higher TRAEs, and (C) immunomodulatory medication use for any-grade TRAE. IO, immuno-oncology; IPI, ipilimumab; NIVO, nivolumab, SSAC, subsequent systemic anticancer therapy.

When TFS was partitioned into health states with and without toxicity, the restricted mean TFS with grade 3 and higher TRAEs was 3% of the 36-month period for nivolumab plus ipilimumab, 1% for nivolumab, and less than 1% for ipilimumab (1.1, 0.5, and 0.2 months, respectively; Fig 5A). The restricted mean TFS without grade 3 or higher TRAEs was 28% of the 36-month period for nivolumab plus ipilimumab, 11% for nivolumab, and 23% for ipilimumab. Patients who received nivolumab plus ipilimumab had longer TFS without grade 3 or higher TRAEs compared with nivolumab and compared with ipilimumab; the restricted mean was 10.1 months for nivolumab plus ipilimumab *v* 4.1 months for nivolumab (difference, 6.0 months; 95% CI, 4.2 to 7.7 months) and 8.5 months for ipilimumab (difference, 1.7 months; 95% CI, −0.4 to 3.6 months; Data Supplement). Pooled across all arms, of the grade 3 or higher TRAEs persisting or newly reported after ICI protocol therapy cessation and, therefore, contributing to TFS with toxicity, 30% had system organ class categorized as gastrointestinal, 5% as hepatic, 5% as pulmonary, 1% as renal, 3% as skin, and 3% as endocrine (Data Supplement); an additional 33% of TRAEs were laboratory test–based AEs categorized as investigations.

With a broader characterization of toxicity, the restricted mean TFS with grade 2 or higher TRAEs was 12% of the 36-month period for nivolumab plus ipilimumab, 4% for nivolumab, and 4% for ipilimumab (4.3, 1.5, and 1.6 months, respectively; Fig 5B). The restricted mean TFS without grade 2 or higher TRAEs was also longer for patients who received nivolumab plus ipilimumab than for those who received nivolumab (difference, 3.7 months; 95% CI, 2.3 to 4.9 months; 19% and 9% of the 36-month period, respectively) and was similar to ipilimumab (difference, −0.2 months; 95% CI, −1.7 to 1.2 months; each 19% of the 36-month period; Data Supplement). Inclusion of grade 2 TRAEs increased the proportions of skin and endocrine TRAEs that contributed to TFS with toxicity (Data Supplement). Similarly, the restricted mean TFS with immunomodulatory medication use was 13% of the 36-month period for nivolumab plus ipilimumab, 3% for nivolumab, and 4% for ipilimumab (Fig 5C). TRAEs that contributed to TFS with immunomodulatory medication use are summarized in the Data Supplement.

## DISCUSSION

The introduction of single-agent and combination ICI therapy for patients with advanced melanoma has contributed to an improvement in OS from a median of 6 to 9 months^3^ to more than 4 years.^4,6,10^ Combination ICI therapy with nivolumab plus ipilimumab significantly prolonged PFS and OS and increased the objective response rate versus ipilimumab alone in phase II^7,8^ and phase III^5,6^ trials, which has led to various regulatory approvals. After a minimum follow-up of 36 months, PFS and OS were numerically improved in the nivolumab plus ipilimumab group compared with the nivolumab group, whereas TRAEs and discontinuations as a result of TRAEs were more frequent.^6^ Although these conventional end points of PFS and OS guide regulatory approvals and clinical decisions, they may not provide a comprehensive assessment of the unique outcomes seen with ICIs.

To more comprehensively capture the experience of every patient, we defined a novel outcome measure, TFS, as the area between the two Kaplan-Meier curves for time to cessation of ICI protocol therapy and time to subsequent systemic anticancer therapy initiation or death (analogous to time to treatment failure and time to second-line therapy, respectively).^14,15^ TFS further integrated the possibility of persisting and/or late adverse effects of initial ICI therapy while free of subsequent therapy. In the pooled CheckMate 067 and 069 data set, patients who received nivolumab plus ipilimumab had longer overall restricted mean TFS (11.1 *v* 4.6 months; 31% *v* 13% of the 36-month period alive and free of subsequent therapy) and longer restricted mean TFS without toxicity (28% *v* 11% of 36-month period alive and free of subsequent therapy and grade 3 or higher TRAEs) than those who received nivolumab. The shorter restricted mean ICI protocol therapy duration in the nivolumab plus ipilimumab group and longer restricted mean time until subsequent systemic therapy initiation were largely the result of early combination ICI therapy cessation for toxicity followed by a treatment-free interval. Whether treatment could have been stopped earlier in other patients who received nivolumab plus ipilimumab or nivolumab, thereby extending their TFS without compromising their survival, remains conjectural but is supported by recent data.^16,17^

As ongoing clinical trials aim to improve OS through combination therapy, ICI-based approaches offer the potential of achieving the patient’s goal of durable remission in the absence of continuous therapy.^3,4,18^ This objective traditionally has been evaluated by the objective response rate (particularly complete response rate) and duration of response among responders as complementary to OS. The maximization of the percentage of patients who achieve a durable response remains a focus of cancer immunotherapy and a justification for use of combination therapy regimens. The induction of a durable response with a shorter amount of therapy could be considered another objective unique to ICI-based combination therapy because it has the potential to decrease the financial cost of treatment^19,20^ and return patients to a noncancer quality of life. The TFS analysis approaches integrated OS with a comparison of how that time was spent for each treatment regimen, under the assumption that quality of life varies while on or off anticancer therapy and with or without toxicity of anticancer therapy. In this example of advanced melanoma, where the observed improvement in OS was somewhat modest for combination nivolumab plus ipilimumab versus nivolumab monotherapy, the TFS analysis characterized the potential for remaining treatment free against the potential for undesirable persistent or late treatment adverse effects with each regimen. Patients who received nivolumab plus ipilimumab had marginally longer restricted mean TFS with grade 3 or higher TRAEs than patients who received nivolumab (1.1 *v* 0.5 months; difference, 0.6 months; 95% CI, 0 to 1.1 months), although the TFS with toxicity represented only approximately 10% of the overall TFS of each treatment group.

We propose that the analysis of TFS could be part of the future reporting of clinical trials that involve immuno-oncology agents compared with one another as well as compared with chemotherapeutic and targeted therapies. TFS might be considered a primary or secondary objective of future trials, particularly those that explore combination regimens with cessation of therapy after a fixed or maximal duration (eg, 2 years) or cessation after achievement of a clinical milestone such as a complete or near-complete response (eg, Optimized Management of Nivolumab Based on Response in Patients With Advanced Renal Cell Carcinoma; ClinicalTrials.gov identifier: NCT03203473) or when the trade-offs of differing doses are under investigation.

A strength of the approach is the inclusion of all patients in the analysis rather than a subset of patients defined by a postrandomization outcome, such as the subset of responders, in isolation. We thereby characterize a well-defined population from therapy initiation.^21,22^ It reflects the multiple facets of patients’ experiences, including the frequency and timing of discontinuation and re-initiation of therapy, by adapting the established quality-adjusted time without symptoms or toxicity (Q-TWiST) methodology,^11,12,23^ which partitions patients’ life experiences over a fixed period into regions that differ with respect to survival, disease control, treatment administration, and toxicity. The approach quantifies areas under Kaplan-Meier curves and compares different treatments by using the restricted mean survival time, which may better capture the time-to-event end point distributions of immuno-oncology regimens than the median or milestone estimates.^11,24,25^

The analyses presented here have limitations. The CheckMate 067 and 069 trials specified a fixed-duration treatment of ipilimumab (four doses) and treatment until disease progression for nivolumab plus ipilimumab and nivolumab, which provides the context for clinical interpretation and comparison of TFS between treatment groups. TFS estimates may be different if maximal nivolumab duration was 2 years. However, the placebo-controlled design illustrates the utility of the analysis to compare shorter ICI regimens aimed at maximizing TFS without compromising OS.^16,17^ The approach did not adequately take into consideration a decrement in OS in its assessment of quantity and quality of life spent in the health states. Patients assigned to ipilimumab had a shorter restricted mean OS than those assigned to nivolumab plus ipilimumab (21.4 *v* 25.7 months; difference, −4.3 months), but the difference in overall TFS was smaller (−2.4 months) because of the fixed four-dose ipilimumab duration, with a correspondingly greater time after subsequent therapy (5.8 months). The utility-weighted feature of the Q-TWiST analysis may be preferred to estimate and compare the restricted mean time to event and is being considered for future analyses. The utility of the TFS approach versus conventional PFS and OS analyses should be assessed. Because this study was focused on the treatment-free period, toxicity during ICI protocol therapy was not incorporated but could be, as in the traditional Q-TWiST. Future iterations also could partition survival after subsequent therapy initiation to characterize times on and off second-line therapy. Finally, the TFS concept assumes that quality of life varies while on and off anticancer therapy and is better during TFS without toxicity. In CheckMate 067, quality of life returned to baseline at 2 to 3 months after discontinuation of nivolumab plus ipilimumab or nivolumab for AEs.^26^ Determination of the extent to which quality of life returned to baseline during TFS and differed with and without toxicity is also worthy of investigation and would provide greater insight into a functional definition of toxicity for integration into the analysis.

In conclusion, in addition to the conventional end points of PFS and OS, clinical trials that involve immuno-oncology agents should estimate and compare TFS with and without toxicity between different therapeutic strategies to capture patient experiences more completely. In patients who receive first-line therapy for advanced melanoma in the CheckMate 067 and 069 trials, TFS in patients who received nivolumab plus ipilimumab was greater than nivolumab or ipilimumab alone, and persistent grade 3 and higher TRAEs made up a small proportion of the TFS period for all treatments. As development of the TFS model advances, it should facilitate the unified analysis of efficacy, toxicity, quality of life, and cost to ensure that we identify treatments that provide the most value for our patients.
